# Royal jelly acid: preparation, metabolism and therapeutic potential

**DOI:** 10.3389/fphar.2025.1561351

**Published:** 2025-05-26

**Authors:** Dandan Zhi, Xiying He, Yunfei Xue, Wenzheng Zhao, Xueyang Gong, Yulong Guo, Xinming Luo, Yakai Tian, Kun Dong

**Affiliations:** ^1^ Yunnan Provincial Engineering and Research Center for Sustainable Utilization of Honey Bee Resources, Eastern Bee Research Institute, College of Animal Science and Technology, Yunnan Agricultural University, Kunming, China; ^2^ First clinical medical College, Yunnan University of Chinese Medicine, Kunming, China; ^3^ Agricultural and Rural Service Center in Wangjiafan Town, Yichang, Hubei, China

**Keywords:** royal jelly acid, 10-HDA, fatty acids, function, pharmacological effects

## Abstract

Royal jelly acid (10-HDA), an unsaturated fatty acid unique to royal jelly, exhibits a diverse range of biological activities, including hypoglycemic, anti-inflammatory, and anticancer properties. In recent years, the increasing demand for natural product supplements as health enhancers has led to a significant rise in the consumption of royal jelly products for daily wellness. Consequently, the market for royal jelly supplements has expanded considerably. Understanding the mechanisms underlying the biological activities of royal jelly acid is crucial for optimizing its therapeutic applications and guiding the development of innovative royal jelly-based products. This review consolidates current research on the preparation, metabolism and potential pharmacological roles of royal jelly acid in managing cancer, inflammatory disorders, and glucolipid metabolic diseases and explores the molecular mechanisms driving these effects. Future research should leverage advanced analytical techniques to uncover the intricate mechanisms of royal jelly acid’s actions, paving the way for its broader integration into healthcare and clinical settings.

## 1 Introduction

**FIGURE 1:**
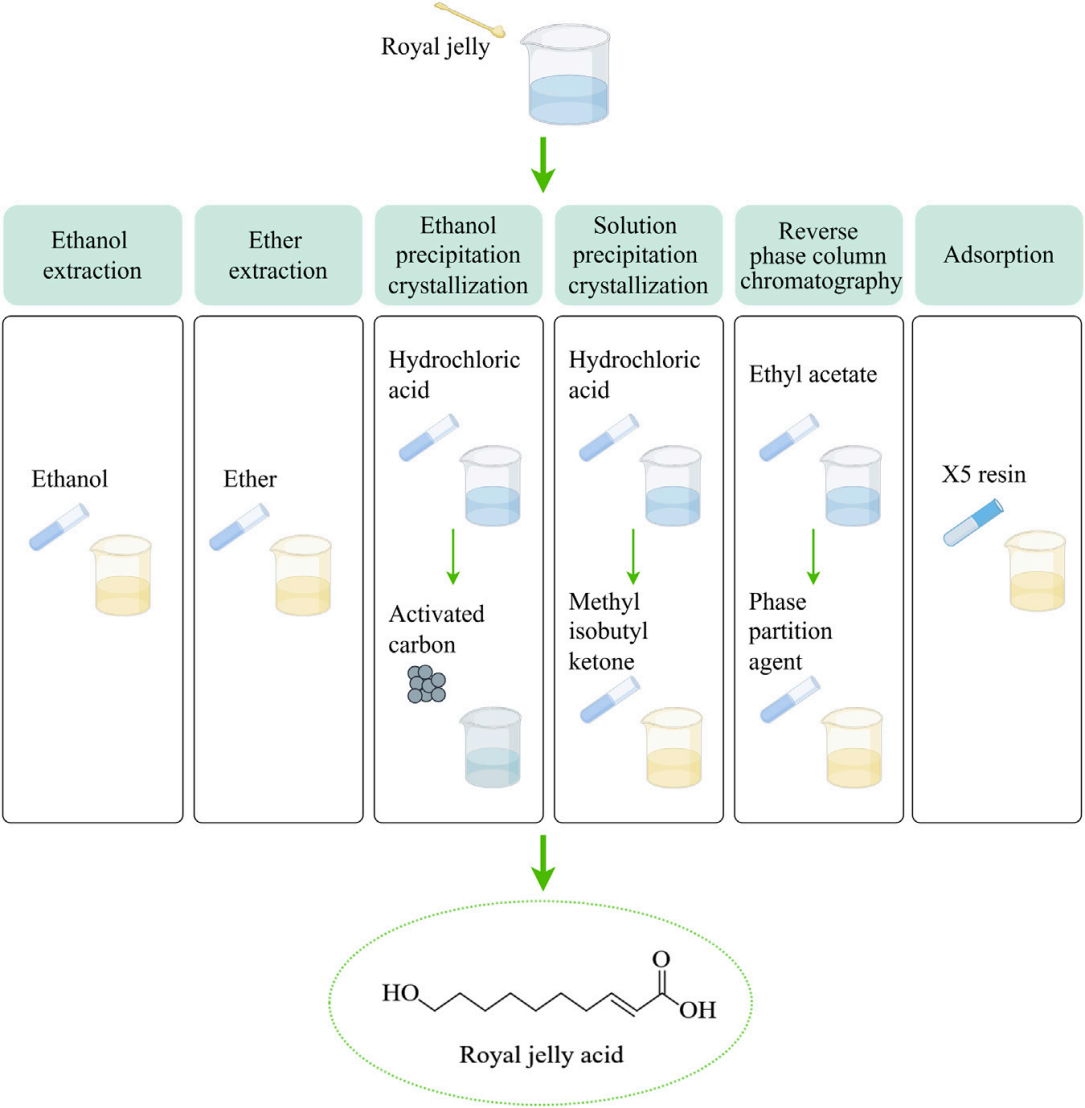
Six methods are used to extract royal jelly acid from royal jelly. In the ethanol extraction method, The lyophilized royal jelly is dissolved in ethanol, and centrifuged, the supernatant is collected and subjected to reverse extraction, crystallization, and drying to isolate royal jelly acid. The ether extraction method builds upon the ethanol extraction method by introducing ether to enhance the extraction process and isolate royal jelly acid more effectively. In the ethanol precipitation crystallization method, first, royal jelly is dissolved in ethanol and centrifuged to obtain the supernatant. The supernatant is acidified with hydrochloric acid, filtered, and dissolved in boiling ethanol. Activated carbon is then added, followed by cooling and crystallization to isolate royal jelly acid. Based on the ethanol precipitation crystallization method, the solution precipitation crystallization method involves melting the solution acidified with hydrochloric acid to form methyl isobutyl ketone. The subsequent steps, including crystallization, follow the ethanol precipitation process. In the reverse phase column chromatography method, first, royal jelly is fractionated using ethyl acetate. ethyl acetate fraction is dissolved in dichloromethane and methanol, the dried and precipitation crystallization in reverse phase column chromatography. In adsorption method, X-5 resin is used to selectively adsorb royal jelly acid from the royal jelly solution. Created by Figdraw.

Natural products have long captured the interest of scientists due to their potent therapeutic benefits and relatively low risk of side effects (Atanasov, et al., 2021). Among these, royal jelly acid—scientifically known as 10-hydroxy-trans-2-decenoic acid (10-HDA, Figure 1)—stands out as a bioactive unsaturated fatty acid with the chemical structure HO-CH2-(CH2)6CH = CH-COOH (C10H18O3), is a unique unsaturated fatty acid found in royal jelly (Hattori, et al., 2007). It is a primary active compound in royal jelly and plays a pivotal role in regulating genetic pathways associated with its biological effects (Spannhoff, et al., 2011). Numerous studies have highlighted the substantial pharmacological potential of royal jelly acid, including its ability to regulate glycolipid metabolism, exert anti-inflammatory effects, and offer neuroprotection (Yu, et al., 2023). Additionally, with its antimicrobial properties (Melliou and Chinou, 2005; Šamšulová, et al., 2023), royal jelly acid has been shown to inhibit a wide range of bacterial strains, making it a promising candidate for combating microbial infections. These attributes have prompted its exploration as a treatment for various conditions, including diabetes (Hu, et al., 2022), colitis (Huang M. et al., 2022), and cancer (Lin, et al., 2020). This review systematically synthesizes research advancements in the preparation methods, metabolic pathways, and therapeutic applications of royal jelly acid through a comprehensive analysis of data curated from multiple scientific databases. It also examines the underlying mechanisms that mediate its pharmacological effects, offering valuable insights into its potential as a treatment for various diseases. Moreover, it underscores the importance of incorporating royal jelly acid into daily healthcare regimens and calls for further investigation into its biological functions and therapeutic potential.

## 2 Preparation methods

### 2.1 Extraction methods

Royal jelly acid can be extracted from royal jelly through several methods, including ethanol extraction (Iegaki, et al., 2020), ether extraction (Antinelli, et al., 2002), ethanol precipitation crystallization (Wang, et al., 2006), solution precipitation crystallization (Wang, et al., 2006), reverse phase column chromatography (Pandeya, et al., 2019) and adsorption method (Zhang and Yang, 2014). The extraction methods are shown in Figure 1. The extraction efficiency of ethanol extraction, ether extraction, ethanol precipitation crystallization and solution precipitation crystallization is generally inefficient, which leads to their gradual phase-out. Reverse phase column chromatography method and adsorption method have garnered attention because of their efficiency and scalability. The X-5 resin demonstrates an adsorption capacity of 9.7 mg/g, with a purity level of approximately 86% (Zhang and Yang, 2014). Its reusability makes this method particularly promising for large-scale industrial applications.

### 2.2 Synthesis methods

Royal jelly acid can also be synthesized using chemical synthesis and microbial synthesis. Additionally, it is naturally produced in the mandibular glands of worker bees, conclude hydroxylation of stearic acid at the 18th (w) or 17th (o-1) position, then β-oxidation to obtain royal jelly acid (Plettner, et al., 1996). The process thought to be closely linked to the *KAT* gene (Liu, et al., 2016).

#### 2.2.1 Chemical synthesis method

Six primary chemical synthesis methods are used for royal jelly acid: Wittig reagent alkylene synthesis, ozonolysis, bromination-elimination alkylene synthesis, Knoevenagel condensation, and increased carbon chain elongation synthesis. The principles or processes of these methods are summarized in Table 1. Although these chemical synthesis methods are well-studied, their industrial application remains limited due to several challenges, including generation of multiple by-products, purification complexity, and relatively low yields.

**TABLE 1:** Chemical synthesis methods of royal jelly acid.

Methods	Raw material	Mechanism	Yield (%)	References
Wittig reagent alkylene synthesis	8-acetoxyoctanal	The Wittig reagent effectively addresses issues related to cis-trans isomerism and double bond isomerism in alkene reactions	-	Fray, et al. (1961a)
	1,8-octanediol	Acylation, oxidation, Wittig-Horner reaction, alkaline hydrolysis, and acidification	80	Zong and Wu (2014)
	8-hydroxyoctanal	Reaction with the Wittig-Horner reagent in the presence of a phase transfer catalyst	77	Villiéras, et al. (1985)
Oxydic ozone synthesis	Cyclic dimer of butadiene	Oxidation, acetylation, hydrolysis, hydrogenation, acetal formation, esterification, reduction, and enylation	49	Odinokov, et al. (1983)
	Oleic acid	The key intermediate methyl dimethyl acetal pelanoate is formed	15	Kharisov, et al. (2002)
Bromination elimination alkylene synthesis	10-acetoxy-decanoic acid	Hell-Vothard-Zelinsky reaction	9.7	Fray, et al. (1961b)
	Undecylenic acid	Hilditch reaction	-	Robert (1960)
Knoevenagel condensation synthesis	1,8-suberic acid	Reduction, oxidation, acetylation, condensation, and hydrolysis	63	Quan, et al. (1992)
	Oleic acid	The key intermediate, 8-oxooctyl acetate, is formed	90	Feng, et al. (2016)
	1,6-hexandiol	Bromination, oxidation, Grignard reaction, and Knoevenagel condensation	75.3	Li, et al. (2007)
Increased carbon chain synthesis	Hexamethyleneglycol	Monochlorination and Grignard reaction	50	Kenneth and Sommers (1948)
	Hydrocyanic acid	Aldehyde reaction	-	Ishmuratov, et al. (2002)

The synthesis methods of royal jelly acid are categorized into six distinct classes based on their underlying chemical principles, and describe its main mechanism.

#### 2.2.2 Microbial synthesis method

Microbial synthesis has recently emerged as a promising alternative for producing royal jelly acid, overcoming some of the limitations associated with traditional production methods (Li, et al., 2022). This approach primarily involves the use of *Escherichia coli* (Fang, et al., 2024; Li, et al., 2022; Wang, et al., 2022) and *Pichia pastoris* GS115 (Zhang and Yang, 2014) as microbial systems. The first method begins with the use of *E. coli* to synthesize royal jelly acid from decanoic acid. The mechanism involves the conversion of decanoic acid into decanoyl-CoA by acyl-CoA synthetase. Subsequently, decanoyl-CoA is transformed into trans-dec-2-enoyl-CoA by acyl-CoA dehydrogenase. The intermediate is further converted into trans-2-decenoic acid through the action of acyl-CoA thioesterase. Finally, the hydroxylation of trans-2-decenoic acid at the terminal position, catalyzed by the P450 enzyme, results in the production of 10-hydroxy-2-decenoic acid (royal jelly acid) (Li, et al., 2022). The second method involves the catalytic conversion of 10-hydroxycapric acid into royal jelly acid by using the yeast species *P. pastoris* GS115 (Zhang and Yang, 2014).

## 3 Metabolic pathways

**FIGURE 2:**
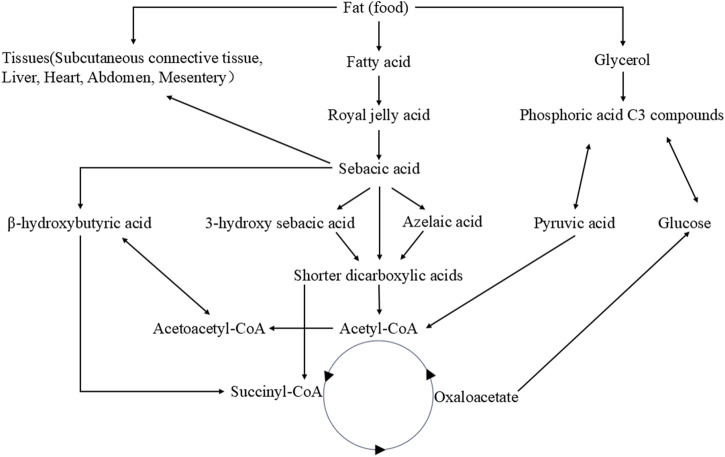
Metabolic pathways of royal jelly acid. The food-derived royal jelly acid is initially oxidized via β-oxidation and ω-oxidation pathways to yield SA. Subsequently, sebacic acid metabolic conversion into shorter-chain dicarboxylic acids. These intermediates are further metabolized through acetyl-CoA to enter the tricarboxylic acid (TCA) cycle, where they undergo complete oxidation to CO_2_ and H_2_O, accompanied by substantial energy release. Created by Figdraw.

Fat metabolism plays a crucial role in maintaining homeostasis within the body (Hansen, et al., 2013). Royal jelly acid undergoes a series of metabolic transformations. Initially, it is metabolized into sebacic acid (SA), which exhibits various physiological activities (Liao, et al., 2024). Subsequently, SA is further broken down into short-chain dicarboxylic acids. These dicarboxylic acids can enter the tricarboxylic acid cycle to release substantial amounts of energy or participate in further metabolic reactions with other bodily substances (Bharathi, et al., 2020). It has been reported that the oxidation of sebacic acid can yield 61 ATP (445.3 kcal/mol), while royal jelly acid may generate over 61 ATP (Liao, et al., 2024). In addition to energy provision, succinate, oxaloacetate, and citrate produced via the tricarboxylic acid cycle play crucial roles in maintaining physiological homeostasis. For instance, citrate synthesis contributes to reduced ketogenesis (Iaconelli, et al., 2010). Furthermore, certain metabolites may continue to release energy through gluconeogenesis (Roden, et al., 2000). Figure 2 presents the metabolic pathways of royal jelly acid. It provides a detailed visualization of royal jelly acid’s metabolic journey.

## 4 Therapeutic potential

Royal jelly acid has demonstrated a broad spectrum of pharmacological effects, including antibacterial activity (Gao, et al., 2022; Šedivá, et al., 2018), regulation of cancer (Saad Al Shehri, et al., 2023), glucolipid metabolic diseases (Hu, et al., 2022), and inflammatory diseases (Huang S. et al., 2022). Studies have recently begun to unravel the molecular mechanisms that underlie these remarkable bioactivities. This section provides a summary of its pharmacological potential.

### 4.1 Anti-bacterial potential

In Gram-positive bacteria, *Paenibacillus larvae*, the causative agent of American foulbrood (AFB), are one of the most devastating bacterial diseases affecting honeybee larvae and pupae (Hansen and and Brødsgaard, 1999). The antibacterial effect of royal jelly acid against *P. larvae* is strongly pH-dependent, with its activity significantly enhanced under acidic conditions (Šamšulová, et al., 2023; Šedivá, et al., 2018). *Staphylococcus aureus* is a common human pathogen responsible for various infectious diseases and a major contributor to foodborne illnesses (Bergin, et al., 2017). Royal jelly acid exhibits a minimum inhibitory concentration of 2.25 mg/mL against *S. aureus* ATCC25923. It inhibits the production of extracellular polymeric substances in *S. aureus* biofilms, prevents biofilm formation. Moreover, royal jelly acid significantly reduces the hemolytic activity of *S. aureus* (Gao, et al., 2022). Royal jelly acid has demonstrated antimicrobial activity against a range of Gram-positive bacteria (Melliou and Chinou, 2005; Yang, et al., 2018), including a cause of sepsis and meningitis, *Streptococcus alactolyticus* (Mylonas, et al., 2020), a cause of skin abscesses, *S. intermedius B* (Biberstein, et al., 1984), linked to various infections, *Staphylococcus xylosus* (Battaglia and Garrett-Sinha, 2023), and a major contributor to dental plaque formation, *Streptococcus mutans* (Forssten, et al., 2010). These findings collectively underscore that royal jelly acid’s potent antibacterial activity, particularly against Gram-positive bacteria, making it a promising candidate for addressing bacterial infections in both humans and animals.

Royal jelly acid demonstrates strong inhibitory effects against several Gram-negative bacteria. *Salmonella choleraesuis*, a pathogen causing systemic infections and potentially fatal mycotic aneurysms (Chiu, et al., 2004), *Vibrio parahaemolyticus*, a significant contributor to acute gastroenteritis in humans (Letchumanan, et al., 2014), and *Escherichia coli (hemolytic)*, a strain associated with bacterial pathogenicity (Griffin and Tauxe, 1991), are all strongly inhibited by royal jelly acid (Yang, et al., 2018). Additionally, *Salmonella typhimurium* induces congestion, edema, and bleeding in the digestive tract (Dar, et al., 2017) and is effectively inhibited by royal jelly acid. Notably, the diHDA-glycerol derivative of royal jelly acid exhibits substantial nonspecific resistance against *S. typhimurium* (Pollet, et al., 2002). Despite these findings, research on the antibacterial effects of royal jelly acid against a broader range of pathogenic Gram-negative bacteria remains limited. Future studies should focus on exploring its potential against diverse pathogenic bacterial strains and pathogenic microorganisms.

Royal jelly acid also exhibits antifungal properties, effectively inhibiting the growth of various pathogenic fungi. *Candida tropicalis* is an opportunistic pathogen causing infections under immunocompromised conditions or altered vaginal environments (Chai, et al., 2010). *Candida albicans* is responsible for infections of the skin, mouth, mucosa, and internal organs (Henriques and Silva, 2021). *Candida glabrata* causes significant systemic infections (Duggan and Usher, 2023). Royal jelly acid inhibits the growth of these pathogenic fungi (Melliou and Chinou, 2005). By contrast, *Saccharomyces cerevisiae*, widely used in the production of bread, wine, and other food products (Parapouli, et al., 2020), is unaffected by royal jelly acid, which shows no inhibitory activity against this yeast (Wang, 2008).

### 4.2 Anti-pest potential

Royal jelly acid demonstrates notable anti-pest properties, targeting a range of vectors and parasites. *Aedes aegypti* is a primary vector for several significant diseases, such as malaria, dengue fever, chikungunya, and Zika virus (Matthews, 2019). *Plasmodium falciparum* is the most common and deadly malaria-causing parasite (Maier, et al., 2019). *Leishmaniasis* is a tropical infection caused by the parasite *Leishmania major* (Sacks and Noben-Trauth, 2002). Royal jelly acid effectively inhibits *Aedes aegypti*, *P. falciparum* K1-strain, and *L. major amastigotes*. The median lethal concentrations for these three pathogens were 31.4, 2.41, and 3.8 μg/mL, respectively. Royal jelly acid exhibited no toxicity to normal human HEK239T cells, suggesting its safety for human applications (Alkhaibari and Alanazi, 2022).

### 4.3 Anti-cancer potential

Royal jelly acid has shown promising anticancer effects in liver, lung, colon, and other types of cancer. In liver cancer research, royal jelly acid significantly inhibited ANGPTL8 expression in HepG2 liver cancer cells (Inoue, et al., 2022). HepG2 cells viability were markedly reduced in a dose-dependent matter. Additionally, it promoted apoptotic pathways by increasing the gene expression of *Caspase-3, Bax* and *miR-34a*, increasing the protein expression of Caspase-3, PARP, and Bax (Saad Al Shehri, et al., 2023). In another experiment, royal jelly acid markedly reduced Hep3B and HCCLM3 liver cancer cell viability, migration and proliferation, and increased the apoptosis process. Multi-omics analysis determined that royal jelly acid interferes with glycolytic metabolism pathway by reducing lactate production, providing a good antitumor effect in both *in vitro* and *in vivo* models (Xu, et al., 2023).

In lung cancer, royal jelly acid inhibited the proliferation of A549, NCI-H460 and NCI-H23 human lung cancer cells, causing nonsignificant toxicity to normal cells. It caused apoptosis in A549 cells through increased the levels of reactive oxygen species. and causes cell cycle arrest at the G0/G1 phase in a time-dependent manner. Furthermore, royal jelly acid modulated key signaling molecules, including increased levels of phosphorylated c-Jun N-terminal kinase (p-JNK), p-p38, and I-κB, whereas reducing the levels of phosphorylated extracellular signal-regulated kinase (p-ERK), p-STAT3, and NF-κB. Moreover, royal jelly acid regulated TGF-β1, SNAI1, GSK-3β, E-cadherin, N-cadherin, and vimentin, inhibiting lung cancer cell migration (Lin, et al., 2020). It suggests that royal jelly acid could potentially be used to treat human lung cancer.

In HCT-116 and SW-480 human colon cancer cells, royal jelly acid has displayed remarkable migratory and invasive effects by inducing significant suppression of promigratory/proinvasive markers, N-cadherin, vimentin and Snail on protein and gene levels (Jovanović, et al., 2022). In WiDr human colon cancer cells, the growth was inhibited by royal jelly acid, and it exhibited anti-inflammatory properties by decreasing NF-κB, IL-8, IL-1β, and TNF-α, and activating IL-1ra (Yang, et al., 2018). Moreover, a combination treatment of royal jelly acid and royal jelly significantly inhibited the growth of CaCo-2 human colon cancer adenocarcinoma cells with decreasing the levels of glutathion and increasing malondialdehyde (Filipič, et al., 2015). While these findings are promising, further *in vitro* studies are needed to confirm the efficacy of royal jelly acid against colorectal cancer.

In Ehrlich solid tumors, royal jelly acid, either alone or when combined with cyclophosphamide, significantly reduced tumor volume and increased the inhibition rates in mice. The treatment also decreased the expression levels of alpha-fetoprotein and carcinoembryonic antigen tumor, tumor necrosis factor alpha, tumor lipid peroxidation, nitric oxide. Furthermore, it enhanced the activity of antioxidant enzymes, including glutathione peroxidase, catalase enzyme, Superoxide dismutase and the apoptotic genes *Caspase-3* and *Bax* (Albalawi, et al., 2021). Additionally, royal jelly acid may also act as a toll-like receptor four antagonist, which could contribute to its tumor-suppressing effects (Eslami-Kaliji, et al., 2022; Eslami-Kaliji, et al., 2021). Research needs to focus on the combination of royal jelly acid.

**FIGURE 3:**
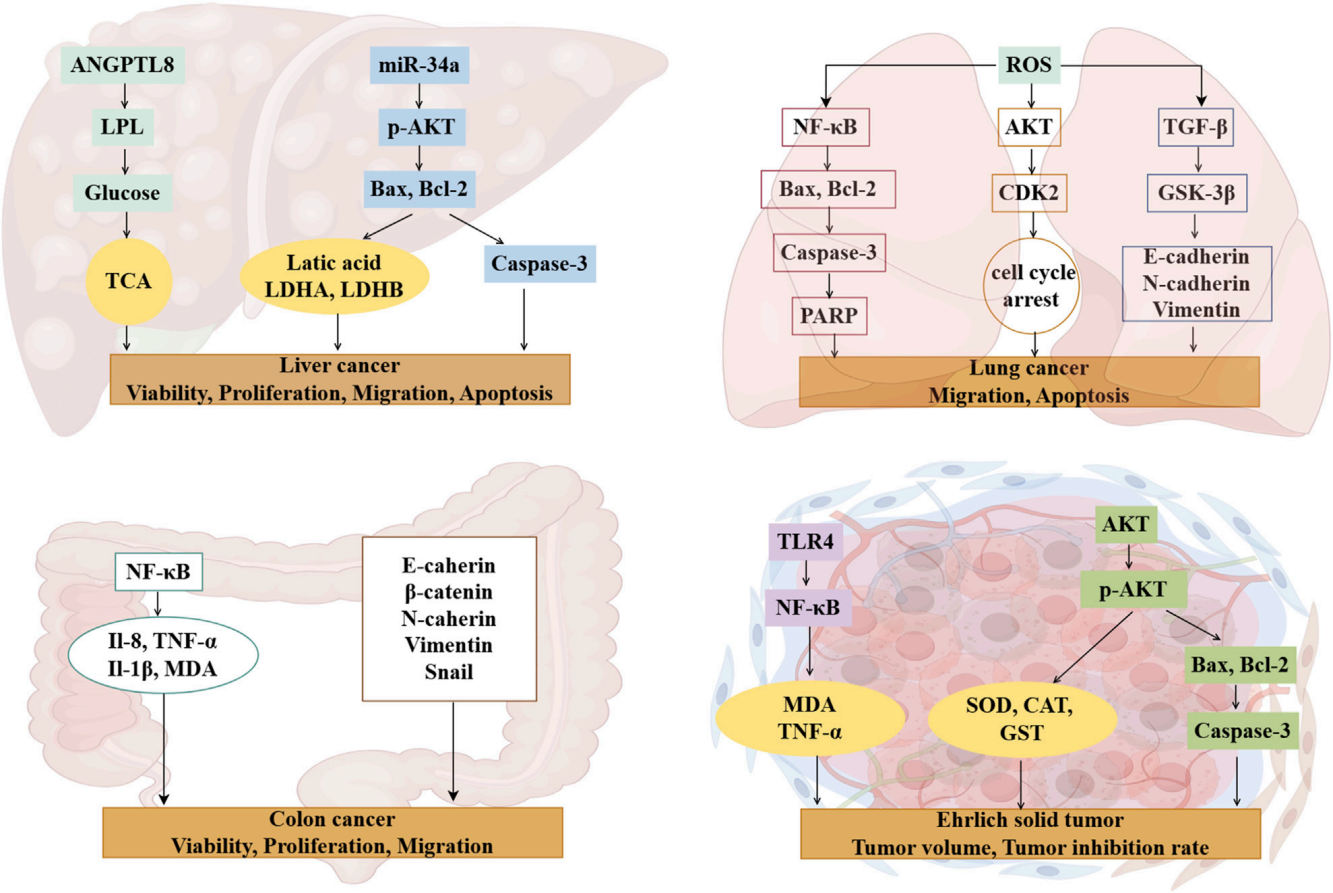
Mechanisms of royal jelly acid on cancer diseases. Royal jelly acid exhibits potential therapeutic benefits against various cancers. In liver cancer, it demonstrates therapeutic potential by modulating key regulators including lactic acid, Caspase-3, and Bax to improve liver cell viability, proliferation, migration, and apoptosis. In lung cancer, royal jelly acid regulates apoptosis-related factors (Bax, Bcl-2, Caspase-3), antioxidant factor (ROS), and anti-inflammatory factor (NF-κB), thereby influencing lung cancer cell migration and apoptosis. For colon cancer, royal jelly acid modulates inflammatory factors (NF-κB, IL-8, IL-1β, TNF-α) and junctional proteins (E-cadherin, β-catenin, N-cadherin), which collectively regulate colorectal cancer cell viability, proliferation, and migration. In Ehrlich solid tumors, royal jelly acid exerts anticancer effects by regulating antioxidant factors (SOD, MDA, CAT) and apoptosis-related factors (Caspase-3, Bax, Bcl-2), leading to reduced tumor volume and enhanced inhibition rate. These findings highlight that royal jelly acid exhibits therapeutic potential against cancer. Created by Figdraw.

Royal jelly acid demonstrated a dose-dependent inhibitory effect on SU-DHL-2 lymphatic cancer cells by modulating key proteins involved in the complement and coagulation cascade pathways, including prothrombin, plasminogen, plasminogen activator, and carboxypeptidyl B2 (Tian, et al., 2024). In HCT116 and MDA-MB-231 breast cancer cells, royal jelly acid reduced histone deacetylase enzyme levels, promoting chromatin lysine residue acetylation and triggering an increase in gene expression levels (Aparecida Dos Santos France, et al., 2024). Moreover, when combined with other royal jelly lipids, royal jelly acid exhibited inhibitory activity on the proliferation of various mammalian cell lines, namely, immortalized C2C12 mouse myoblast cells, PC3 human prostate cancer cells, and SH-SY5Y human neuroblastoma cells. Royal jelly acid also significantly inhibited the proliferation, migration, and tube formation of human umbilical vein endothelial cells (Izuta, et al., 2009), thereby limiting tumor angiogenesis and progression. Ultimately, royal jelly acid has important anti-cancer effects, and more robust *in vitro* experiments are required. The anti-cancer action of royal jelly acid is represented in Figure 3.

### 4.4 Anti-glucolipid metabolic diseases potential

**FIGURE 4:**
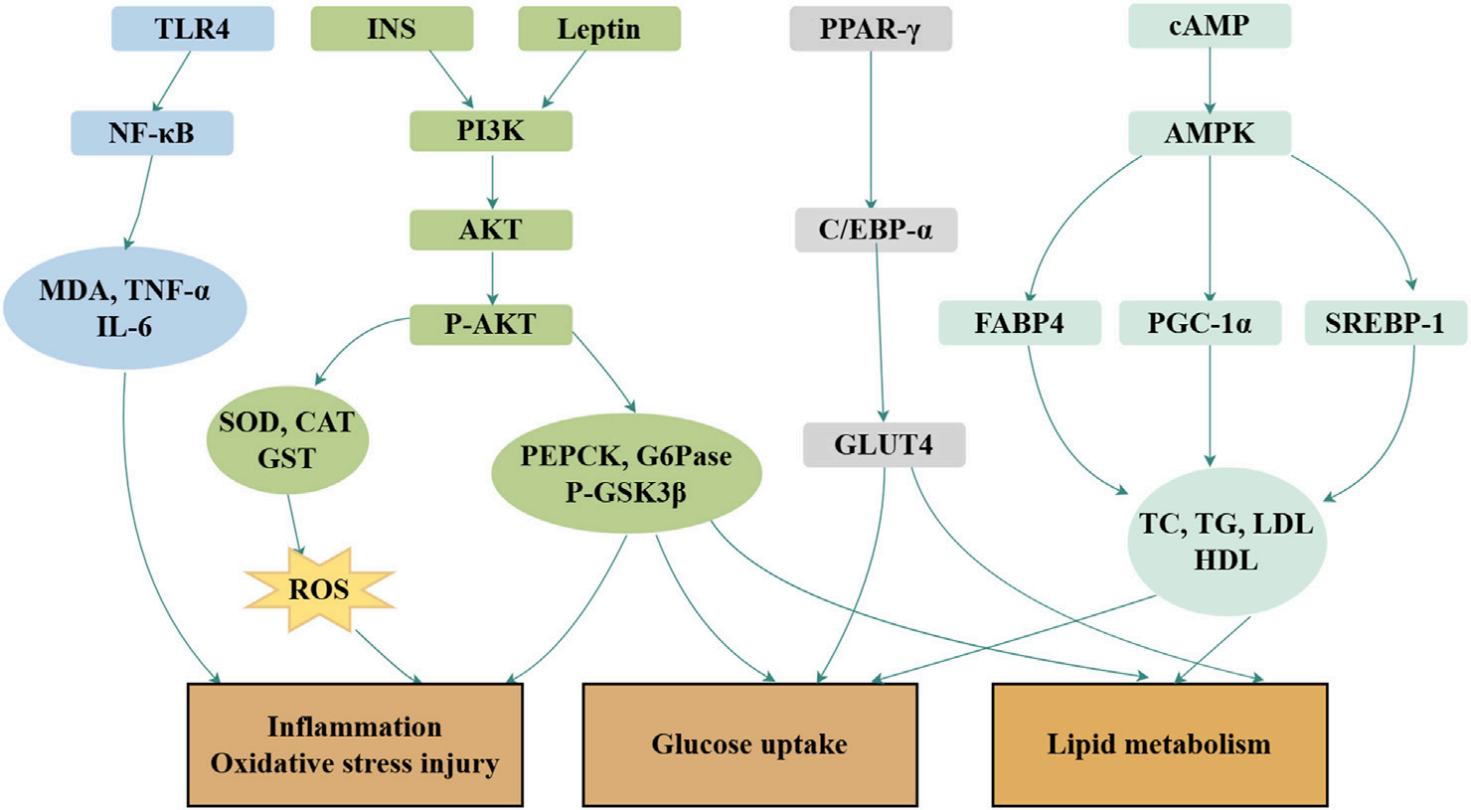
Mechanisms of royal jelly acid on glucolipid metabolic diseases. In glucolipid metabolic diseases, royal jelly acid demonstrates therapeutic potential through multi-target regulatory effects. It modulates lipid factors (TC, TG, LDL, HDL), anti-inflammatory factors (TNF-α, IL-6), and antioxidant factors (SOD, CAT, GST), thereby ameliorating inflammation, oxidative stress injury, glucose uptake, and lipid metabolism dysregulation. Created by Figdraw.

Royal jelly acid exhibits potent therapeutic effects in the treatment of glycolipid metabolic disorders through its regulatory, anti-inflammatory, and antioxidant properties (Hu, et al., 2022). In hyperlipidemic rats, royal jelly acid significantly reduced the levels of triglycerides, total cholesterol, and β-lipoproteins, whereas increased high-density lipoprotein levels (Xu, et al., 2002). A total of 41 key metabolites played key roles in the exertion of antihyperlipidemic effects, including glutamylaspartic acid, caproic acid and tryptophyl-glutamine (Zhi, et al., 2025). In studies involving type 2 diabetes mice, royal jelly acid displayed strong hypoglycemic effects by modulating inflammatory markers such as IL-6 and TNF-α, and by enhancing antioxidant enzymes, including SOD, CAT, and GPx. The compound improved glucose metabolism through activation of the PI3K/AKT signaling pathway and upregulated the expression of PGC-1α, a key regulator of mitochondrial biogenesis in mice (Hu, et al., 2022). In HepG2 liver cells, royal jelly acid increased phosphorylated AMP-activated protein kinase levels, inhibited aquaporin-9 gene expression, and facilitated glycerol uptake by liver tissues (Watadani, et al., 2017). According to animal studies, royal jelly acid enhances pAMPK phosphorylation and facilitates GLUT4 translocation to the cell membrane, thereby improving glucose uptake in skeletal muscle cells (Usui, et al., 2019). In 3T3-L1 adipocytes, royal jelly acid downregulated oxidative stress markers such as ROS and suppressed the expression of key lipogenic transcription factors, including PPARγ, FABP4, C/EBP-α, SREBP-1c, and leptin (Pandeya, et al., 2019). The compound also scavenged DPPH radicals (Kolayli, et al., 2016) and activated TRPA1 and TRPV1 channels, inducing thermogenesis and augmenting energy expenditure (Terada, et al., 2011). Royal jelly acid evidently has a more beneficial effect on glycolipid metabolic disease improvement. The primary mechanism is depicted in Figure 4.

### 4.5 Anti-inflammatory diseases potential

Inflammation plays a pivotal role in the development and progression of various diseases (Okin and Medzhitov, 2012). Lipopolysaccharide (LPS), commonly used to induce immune responses in mammalian cells, triggers the release of pro-inflammatory cytokines, and serves as a standard model for studying inflammation (Zhang, et al., 2024). In LPS-induced inflammation models generated using RAW264 cells, royal jelly acid exhibits anti-inflammatory effects by inhibiting IκB-ζ and IL-6 expression, although it does not influence TNF-α levels (Spannhoff, et al., 2011). By contrast, in another study, royal jelly acid significantly downregulated TNF-α expression while markedly inhibiting IL-6 and IL-1β production (Huang M. et al., 2022). Further research revealed that royal jelly acid alleviates inflammation by suppressing JNK1/2 and p38 MAPK phosphorylation (Chen, et al., 2016), resulting in reduced IL-6 (Chen, et al., 2016; Huang S. et al., 2022; Sugiyama, et al., 2012) and IL-1β (Huang M. et al., 2022) levels. In BV-2 microglial cells exposed to LPS-induced inflammation, royal jelly acid mitigated inflammation by directly inhibiting the NLRP3 inflammasome or indirectly promoting autophagy (You et al., 2020a). Metabolomic and transcriptomic analyses further highlighted that royal jelly acid modulates pathways involved in amino acid metabolism, antigen processing and presentation, and NOD receptor signaling, which are critical to its anti-inflammatory effects (Huang, et al., 2023). Royal jelly acid has exhibited major protective effects against inflammation both *in vitro* and *in vivo*.

Nitric oxide (NO) is a pro-inflammatory mediator that contributes to various inflammatory diseases by generating peroxynitrite anions (Litvinova, et al., 2015). In a model of NO-induced inflammation in RAW264 cells treated with IFN-γ, royal jelly acid significantly reduced NO production and suppressed the activation of the NO synthase promoter. It also inhibited the expression of IRF-8, TNF-α, and NF-κB, while leaving IRF-1 expression unaffected (Takahashi, et al., 2012). Similar findings were observed in another inflammatory RAW264 cell model, where LPS- or IFN-β-mediated NO production was significantly reduced by royal jelly acid, which concurrently increased TNF-α and NF-κB levels (Sugiyama, et al., 2012).

**FIGURE 5:**
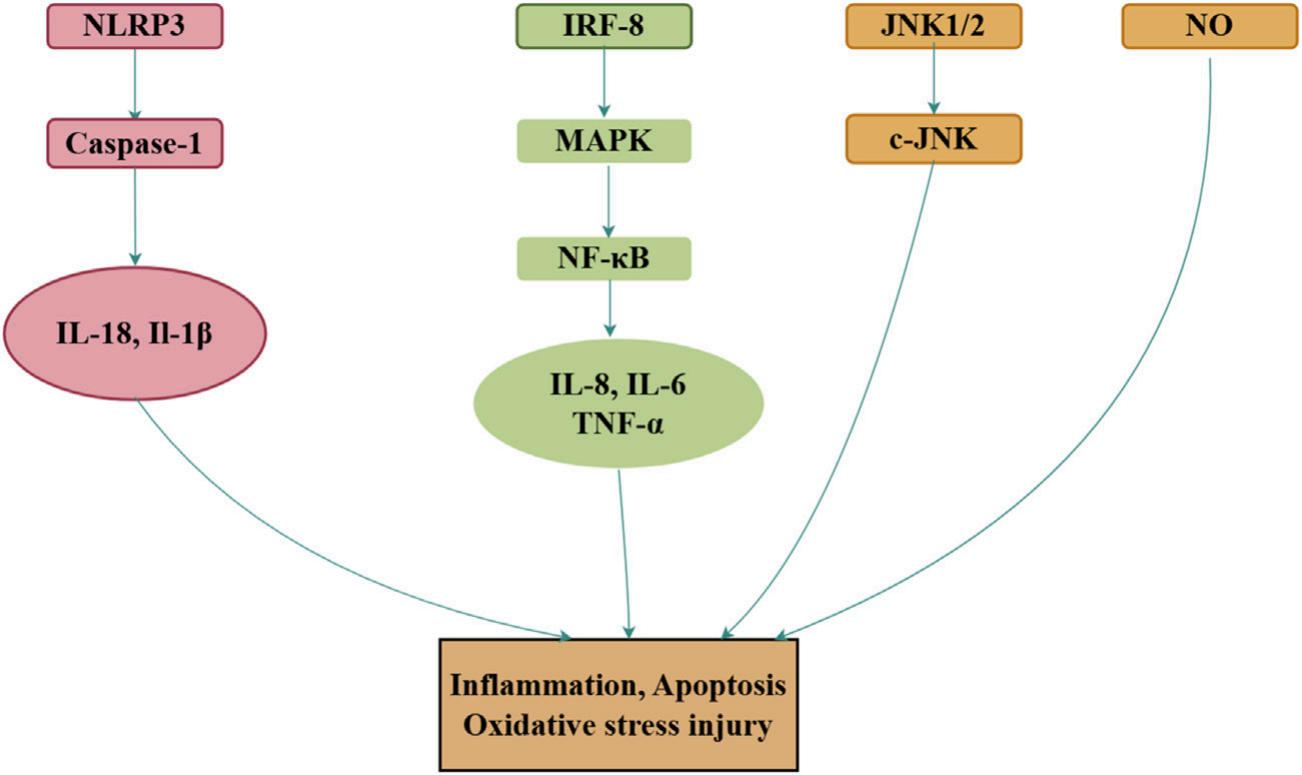
Mechanisms of royal jelly acid on inflammatory diseases. In inflammatory diseases, royal jelly acid demonstrates therapeutic potential by modulating anti-inflammatory factors (TNF-α, IL-17, IL-1β) to regulate inflammation, apoptosis, and oxidative stress injury. Created by Figdraw.

Several *in vivo* studies have further validated the anti-inflammatory properties of royal jelly acid. In a mouse model of colitis, royal jelly acid significantly reduced the expression of key inflammasome components, including TXNIP, NLRP3, ASC, Caspase-1, GSDMD, N-GSDMD, IL-1β, and IL-18, while in THP1 cells, it inhibited LPS/ATP-induced inflammasome-mediated pyroptosis, thereby alleviating inflammation and associated colitis (Huang S. et al., 2022). Additionally, in chicken models of intestinal mucosal injury, royal jelly acid reduced the levels of pro-inflammatory cytokines (TNF-α, IL-1β, and IL-6), reversed the upregulation of TLR4 and NF-κB, and ameliorated intestinal mucosal injury (Han, et al., 2023). In rheumatoid arthritis synovial fibroblast cells, royal jelly acid modulated the p38 kinase and c-Jun N-terminal kinase (JNK)-AP-1 signaling pathways, thereby alleviating symptoms of rheumatoid arthritis (Yang, et al., 2010). Although royal jelly acid has demonstrated effective anti-inflammatory properties *in vitro* and *in vivo*, more studies are necessary to explore its potential mechanisms in different inflammatory and related conditions. Figure 5 illustrates the main mechanism.

#### 4.6 Anti-neurological diseases potential

Royal jelly acid has demonstrated potential neuroprotective and antidepressant effects across various studies (Dubey, et al., 2024; Gong, et al., 2023; Ito, et al., 2012). It enhances neuronal development, especially when used in conjunction with TAU protein, and displays strong binding affinity for TAU protein kinase and phosphatase (Dubey, et al., 2024). Royal jelly acid activates TRPA1 (Terada, et al., 2011), acetylates histones H3 and H4, and inhibits the expression of active caspase-3 and PARP-1 (Koc, et al., 2025), thus contributing to neuroprotection. In microglial BV2 cells, royal jelly acid alleviates neuroinflammation by promoting the FOXO1-mediated autophagy pathway (You et al., 2020b) It also enhances neurogenesis in cultured neural stem/progenitor cells (Hattori, et al., 2007) and preserves blood–brain barrier integrity by inhibiting the degradation of tight junction proteins via the AMPK/PI3K/AKT signaling pathway (You, et al., 2019). Likely in APP/PS1 mouse models of Alzheimer’s disease, royal jelly significantly improved memory deficits by stimulating the cAMP/PKA/CREB/BDNF pathway and reducing neuronal apoptosis (You, et al., 2018). Moreover, when combined with aspirin to treat memory impairment and neuroinflammation, royal jelly acid synergistically reduced pro-inflammatory mediator levels, inhibited glial cell activation, and alleviated both memory deficits and neuroinflammation (You, et al., 2022). Incorporating royal jelly and royal jelly acid as supplements could be a hopeful therapeutic strategy for neurological ailments.

### 4.7 Other beneficial potentials

In life extension and immune enhancement, The queen bee, which eats royal jelly for its entire life, lives about ten times longer than the worker bee. Studies have shown that royal jelly acid, a special fatty acid in royal jelly, can also prolong lifespan and boost immunity in various models. Studies on *Caenorhabditis elegans* revealed that lipid mixtures containing royal jelly acid increased lifespan by activating the FOXO transcription factor DAF-16 and reducing insulin/IGF-1 signaling (IIS) (Honda, et al., 2011). Interestingly, it also extended lifespan through mechanisms IIS-independent mechanisms involving dietary restriction and TOR signaling pathways (Honda, et al., 2015). Additionally, in cyclophosphamide-induced immunocompromised mice, royal jelly acid restored body weight and increased thymus and spleen mass. It enhanced DNA/RNA/protein activity, while stimulating pathways that support B lymphocyte affinity maturation, antigen presentation, and macrophage activity (Fan, et al., 2020). These effects indicate its immune regulatory potential. Furthermore, royal jelly acid inhibited T cell proliferation, downregulated IL-2, CD86, MHC II, and IL-12 production, and upregulated IL-10 production, demonstrating its ability to modulate immune responses effectively (Vucevic, et al., 2007). These properties contribute to its role in improving immunity and addressing immune-related diseases.

Royal jelly acid exhibits protective effects in a range of conditions, including testicular toxicity, osteoporosis, and dry eye disease in mice. In studies on rats exposed to PbAc, royal jelly acid improved testicular function. A combination of royal jelly acid and ZnO nanoparticles reduced inflammatory and apoptotic markers (Caspase-3 and Bax); enhanced semen quality; and improved the levels of pituitary and gonadal hormones, antioxidants, and testicular tissue structure (Maher, et al., 2024). Regarding osteoporosis, royal jelly acid interacts with FFAR4 to inhibit NF-κB signaling, which contributes to its anti-osteoporotic effects in mice (Tsuchiya, et al., 2020). While it did not prevent bone loss following ovariectomy, it improved bone density and reduced femoral bone hardness, suggesting potential benefits in bone health (Hanai, et al., 2023). Moreover, royal jelly acid stabilizes acetylcholine levels, which may help alleviate symptoms of dry eye disease, improving tear production and ocular surface health (Yamaga, et al., 2021). Given the anti-inflammatory, anti-bacterial, and anti-cancer properties of royal jelly acid, more research is needed to understand the specific mechanisms of its effects on various diseases.

## 5 Conclusion

As a natural product, royal jelly acid has exhibited therapeutic potential against various diseases and pathological conditions. Both *in vivo* and *in vitro* studies have demonstrated its pharmacological versatility, but much remains to be explored regarding its efficacy and safety in humans. Key research areas include exploring the pharmacokinetics of royal jelly acid, that is, how royal jelly acid is absorbed, metabolized, and excreted in the human body, and its potential for inducing drug resistance; conducting rigorous studies to evaluate its efficacy and safety in treating specific human diseases, including liver cancer, hyperlipidemia and colitis; elucidating the mechanisms underlying anti-inflammatory, antioxidant, and lipid metabolism-regulating effects of royal jelly acid; exploring age-stratified studies, including adolescents, pregnant individuals, and geriatric cohorts; accelerating the clinical research; assessing the safety profile, including potential side effects and interactions with other drugs or conditions; enhancing bioavailability by developing optimized drug formulations to augment its absorption and therapeutic action; and utilizing advanced technologies such as single-cell sequencing, spatial transcriptomics, and gut microbiome analysis to uncover its molecular pathways. Concomitantly, intensified research efforts should prioritize the investigation of royal jelly acid derivatives, particularly diHDA-glycerol derivative of royal jelly acid. Strengthen the development of royal jelly acid-based functional products, including nutraceutical supplements and bioactive beverages. While royal jelly acid holds significant promise for daily healthcare and therapeutic applications, a comprehensive understanding of its mechanisms, safety, and effectiveness remains a significant challenge for future research.
